# Glymphatic system dysfunction and cerebrospinal fluid retention in gliomas: evidence from perivascular space diffusion and volumetric analysis

**DOI:** 10.1186/s40644-025-00868-y

**Published:** 2025-04-07

**Authors:** Weiqiang Liang, Wenbo Sun, Chunyan Li, Jie Zhou, Changyou Long, Huan Li, Dan Xu, Haibo Xu

**Affiliations:** 1 Department of Radiology, Zhongnan Hospital of Wuhan University, 169 Donghu Road, Wuhan, 430071 China; 2 Department of Nuclear Medicine, Zhongnan Hospital of Wuhan University, Wuhan University, 169 Donghu Road, Wuhan, 430071 China; 3 Hubei Provincial Engineering Research Center of Multimodal Medical Imaging Technology and Clinical Application, Wuhan, China

**Keywords:** Glioma, Glymphatic system, Cerebrospinal fluid retention, Diffusion tensor image analysis along the perivascular space (DTI-ALPS), Magnetic resonance imaging

## Abstract

**Background:**

Gliomas may impair glymphatic function and alter cerebrospinal fluid (CSF) dynamics through structural brain changes, potentially affecting peritumoral brain edema (PTBE) and fluid clearance. This study investigated the impact of gliomas on glymphatic system function and CSF volume via diffusion tensor imaging analysis along the perivascular space (DTI-ALPS) and volumetric magnetic resonance imaging (MRI), which clarified the relationships between tumor characteristics and glymphatic system disruption.

**Methods:**

In this prospective study, 112 glioma patients and 56 healthy controls underwent MRI to calculate DTI-ALPS indices and perform volumetric analyses of CSF, tumor, and PTBE. Statistical analyses were used to assess the relationships between the DTI-ALPS index, tumor volume, PTBE volume, and clinical characteristics.

**Results:**

Glioma patients had significantly lower DTI-ALPS indices (1.266 ± 0.258 vs. 1.395 ± 0.174, *p* < 0.001) and greater CSF volumes (174.53 ± 34.89 cm³ vs. 154.25 ± 20.89 cm³, *p* < 0.001) than controls did. The DTI-ALPS index was inversely correlated with tumor volume (*r* = -0.353, *p* < 0.001) and PTBE volume (*r* = -0.266, *p* = 0.015). High-grade gliomas were associated with lower DTI-ALPS indices and larger PTBE volumes (all *p* < 0.001). Tumor grade emerged as an independent predictor of the DTI-ALPS index in multivariate analysis (β = -0.244, *p* = 0.011).

**Conclusion:**

Gliomas are associated with significant glymphatic dysfunction, as evidenced by reduced DTI-ALPS indices and increased CSF and PTBE volumes. The DTI-ALPS index serves as a potential biomarker of glymphatic disruption in glioma patients, offering insights into tumor-related fluid changes and the pathophysiology of brain-tumor interactions.

**Supplementary Information:**

The online version contains supplementary material available at 10.1186/s40644-025-00868-y.

## Background

The glymphatic system, a recently characterized network, plays a crucial role in maintaining cerebral homeostasis by facilitating the clearance of interstitial waste products and regulating fluid dynamics within the brain [1]. This system operates through a coordinated mechanism in which arterial pulsations propel cerebrospinal fluid (CSF) along the perivascular space (PVS), whereas astrocytic end-feet equipped with aquaporin-4 (AQP4) channels facilitated the influx of CSF into the brain parenchyma, where it is exchanged for interstitial fluid (ISF) and then drains intracerebral solutes through the meningeal lymphatic ducts into the cervical lymphatic system [2–4]. The proper functioning of the glymphatic system is crucial for brain health, as it allows for the clearance of neurotoxic agents and helps maintain the extracellular fluid balance [5, 6]. Studies have shown correlations between disturbances in glymphatic pathways and various neurological conditions, such as Alzheimer’s disease, Parkinson’s disease, and traumatic brain injury [7–10]. However, these associations remain observational, and further research is needed to clarify their causal relationships.

**Fig. 1:**
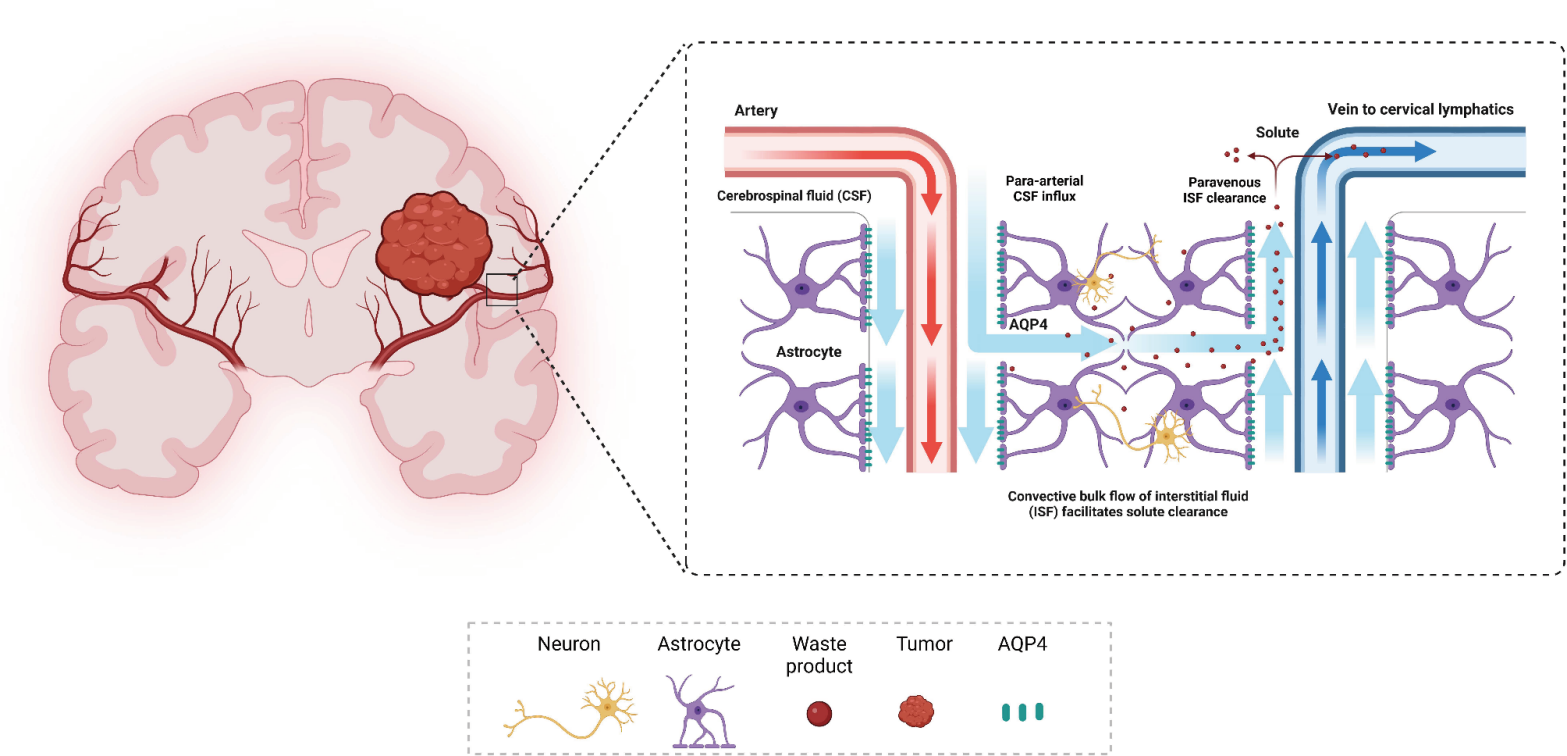
Schematic diagram of the glioma and glymphatic pathways. CSF enters the brain parenchyma along the perivascular space of arterioles, where it undergoes diffusion and convection mediated by AQP-4 to exchange solutes with the ISF. After this exchange, CSF moves towards the perivascular space of the veins and ultimately flows to the meningeal lymphatic system and cervical lymph nodes. Additionally, fluid efflux may occur through other undefined pathways, such as along nerve bundles like the olfactory nerve. CSF, cerebrospinal fluid; ISF, interstitial fluid. (Adapted from an image created by BioRender.com.)

In brain tumors, especially gliomas, the brain undergoes significant structural and functional changes, including elevated intracranial pressure, blood‒brain barrier (BBB) disruption, and white matter fiber bundle damage [11, 12]. However, the potential impact of gliomas on glymphatic function remains inadequately studied. Preliminary animal studies have shown that the interstitial fluid (ISF) clearance time, measured using the tracer Gd-DTPA, is significantly prolonged in AQP4 knockout rats compared to wild-type controls (82.8 ± 6.95 vs. 52.60 ± 6.87 min, *p* < 0.05), which might indirectly reflect localized disturbances in glymphatic activity [13]. This localized glymphatic dysfunction may impede the timely clearance of tumor-derived inflammatory and immune factors and thus affecting tumor cell metabolism and growth. Additionally, in glioma models, AQP4 expression is significantly elevated in the peritumoral region, with a positive correlation between AQP4 overexpression and the severity of peritumoral brain edema [14]. A melanoma mouse model study shows that AQP4-deficient mice exhibit more severe vasogenic edema and increased intracranial pressure compared to wild-type mice [15]. These findings suggest a potential link between AQP4 dysregulation and impaired brain fluid dynamics. Our preclinical studies further indicate that CSF efflux is significantly reduced in glioma models, which may exacerbate peritumoral brain edema (PTBE) and alter the tumor microenvironment, thereby affecting the effectiveness of intratumoral drug delivery [16]. Nevertheless, the intricate relationship between glioma-induced structural and functional changes and glymphatic function remains poorly understood, especially in human studies. This knowledge gap highlights the need for in-depth studies on how these changes affect homeostasis and clinical outcomes in the brain (Fig. 1).

Traditionally, evaluating glymphatic function has depended on invasive methods such as intrathecal tracer delivery or gadolinium-enhanced MRI. While these approaches have yielded valuable insights, their invasiveness and limited temporal resolution reduce their feasibility for routine clinical application [17, 18]. Fortunately, advances in MRI technology have led to promising noninvasive alternatives. The diffusion tensor imaging along perivascular spaces (DTI-ALPS) method has been proposed as a potential surrogate for glymphatic activity, based on its ability to quantify anisotropic water diffusion along PVS [19]. While a lower DTI-ALPS index has been interpreted as a potential marker of glymphatic dysfunction, this relationship requires further validation, as direct evidence linking ALPS indices to interstitial solute clearance in humans is still limited.

This study investigated the impact of gliomas on glymphatic function via noninvasive DTI-ALPS. By analyzing the relationships among the ALPS index, PTBE, and CSF volume, we aimed to clarify how gliomas disrupt brain fluid homeostasis. These findings may inform strategies to restore glymphatic function and mitigate tumor-related complications.

## Materials and methods

### Ethical permissions

This prospective study was conducted in compliance with the Declaration of Helsinki and received authorization from the Ethical Review Committee of our hospital (Approval No. 2020109). All participants provided written informed consent prior to their inclusion in the study.

### Subjects

A total of 112 patients with gliomas (49 females, mean age 52.8 ± 13.1 years) who were treated at the Neurosurgery Department at our hospital between October 2020 and July 2023 were enrolled in this prospective study. Among these patients, 35 had low-grade gliomas, and 77 had high-grade gliomas. Additionally, 56 age- and sex-matched healthy volunteers (27 females, mean age 53.4 ± 10.7 years) were recruited from the hospital’s medical checkup center. Patients who met the following criteria were excluded from the study: (1) absence of histopathological and genetic testing information; (2) presence of other neurological or psychiatric disorders, such as intracranial infections or cerebrovascular diseases; (3) significant sleep dysfunction; and (4) poor MRI image quality. Healthy volunteers were included on the basis of the following criteria: (1) had no known history of neurological or psychiatric disorders; (2) had no abnormal brain structures or lesions detected on MRI scans; (3) were free from systemic diseases that could affect the central nervous system, such as diabetes or hypertension; and (4) were not currently taking medications that could influence central nervous system function.

### MRI acquisition

MRI data were obtained via a 3T scanner (uMR 790, United Imaging Healthcare, Shanghai, China) that featured a 24-channel head coil. DTI was performed with a maximum b value of 1000 s/mm², utilizing 48 diffusion-encoding directions. The parameters of acquisition were as follows: repetition time (TR) = 5,116 ms, echo time (TE) = 74.20 ms, slice thickness = 4 mm, number of slices = 40, field of view (FOV) = 224 × 224 mm², flip angle = 90°, and bandwidth = 1800 Hz/Px. Additionally, a 3D T1-weighted fast gradient echo sequence (3D T1W) was used both before and after the administration of a gadolinium-based contrast agent, gadobutrol (Gadavist, Bayer, Germany), which was delivered intravenously at a dosage of 0.1 mmol/kg via a high-pressure injector with a flow rate of 1.5 mmol/s. 2D T2-weighted fast spin echo images (2D T2W) were obtained to evaluate anatomical features and lesion attributes, whereas a 3D fluid-attenuated inversion recovery (FLAIR) sequence was employed to minimize the signals from CSF, enhancing the visualization of periventricular and cortical lesions. To account for the effect of circadian rhythms on the activity of the glymphatic system, all MRI scans included in this study were scheduled to take place between 9:00 am and 12:00 pm [20]. For more comprehensive details regarding the MRI scan parameters, please consult Supplementary Table 1.

### Image analysis

#### Diffusion tensor image analysis along the perivascular space (DTI-ALPS)

**Fig. 2:**
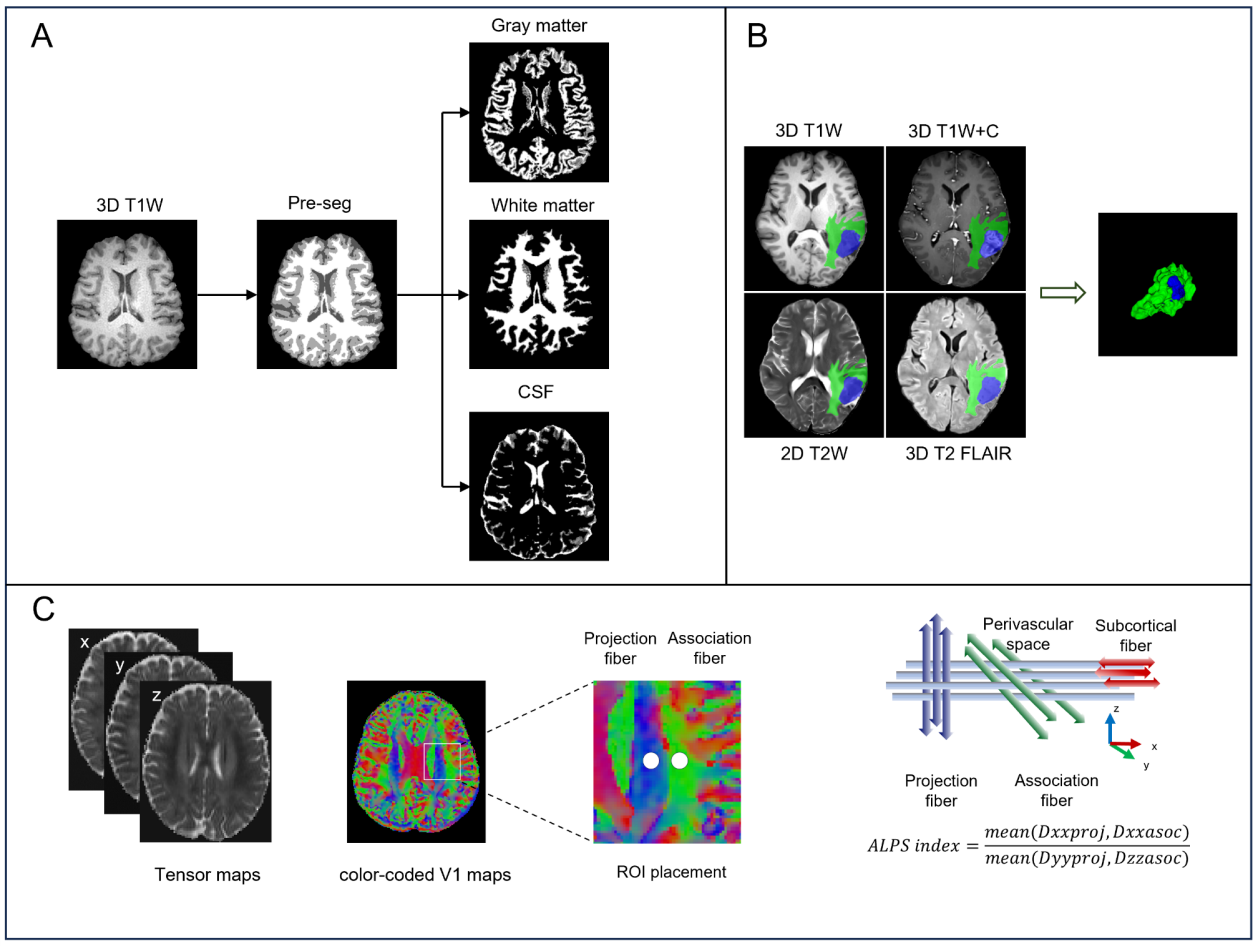
Flowchart of MRI image analysis. (**A**) Segmentation of 3D-T1 images into white matter, gray matter, and CSF via FSL software, followed by calculation of the CSF volume. (**B**) Manual delineation of brain tumor (blue area) and peritumoral edema (green area) VOIs on 3D T1 + C and T2 FLAIR images, respectively. (**C**) Processing flow of the DTI-ALPS index: preprocessing of diffusion tensor images via FSL. Four 4-voxel cubic VOIs were defined bilaterally at the lateral ventricle level to evaluate diffusion coefficients in projection and association fibers

The DTI-ALPS index was used to assess the rate of water diffusion within the PVS surrounding the medullary veins at the level of the lateral ventricles. It serves primarily to indicate the ability of the brain to transport fluid from subcortical regions toward the lateral ventricles and has been utilized to indirectly evaluate overall glymphatic function in the brain [19]. The process of analysis can be summarized in the following steps: (1) Preprocessing of the DTI data was conducted on a Linux workstation with the FMRIB Software Library (FSL, version 6.0.1, University of Oxford, UK, http://www.fmrib.ox). This step included eddy current correction, motion correction, and skull stripping. (2) The diffusion tensor was computed via the DTIFIT tool, which produces color-coded fractional anisotropy (FA) maps along with diffusion coefficients on the x-, y-, and z-axes. (3) Two neuroradiologists outlined four 4-voxel cubic volumes of interest (VOIs) in both the affected tumor hemisphere and the corresponding contralateral hemisphere at the lateral ventricle level, locating these VOIs in the projection and association fiber regions, ensuring placement in areas unaffected by direct tumor invasion (Fig. 2 (**C**)). To ensure consistency, ROI placement was standardized by selecting a fixed plane at the lateral ventricle level, where the projection and association fibers show distinct signal intensity differences on tensor maps, facilitating precise localization. (4) The diffusion coefficient D of the voxel levels within the identified VOIs was statistically analyzed by two radiologists along the x-, y-, and z-axis dimensions, leading to the determination of Dxxproj, Dxxassoc, Dyyproj, and Dzzassoc values, respectively. The mean values from these coefficients were subsequently calculated. The DTI-ALPS index was ultimately established via the following formula:

$$\:\text{D}\text{T}\text{I}-\text{A}\text{L}\text{P}\text{S}\:\text{i}\text{n}\text{d}\text{e}\text{x}=\frac{\text{m}\text{e}\text{a}\text{n}(\text{D}\text{x}\text{x}\text{p}\text{r}\text{o}\text{j},\:\text{D}\text{x}\text{x}\text{a}\text{s}\text{s}\text{o}\text{c})\:}{\text{m}\text{e}\text{a}\text{n}(\text{D}\text{y}\text{y}\text{p}\text{r}\text{o}\text{j},\:\text{D}\text{z}\text{z}\text{a}\text{s}\text{s}\text{o}\text{c})}$$

#### Segmentation of CSF, tumor, PTBE, and volumetric analysis

Unenhanced 3D-T1W images were first registered to the MNI152 standard brain template and skull stripping was then performed using the bet tool in FSL to remove non-brain tissues. Subsequently, the images were divided into cerebral gray matter, cerebral white matter, and CSF via FAST Segmentation, an automated software for FSL, with the volume of CSF being calculated. The volume of interest (VOI) for the tumor, encompassing both enhancing and non-enhancing areas, including necrotic cores, was delineated on the enhanced 3D T1W images. The PTBE was specifically segmented on 3D T2 FLAIR images to accurately capture the extent of edema (Fig. 2 (**A**)). First, the 3D-T1W, 3D-T1W + C, 3D-T2-FLAIR, and 2D-T2W images were aligned through linear registration techniques in SPM12, implemented in MATLAB (R2016b, The MathWorks, Inc.). Two experienced neuroradiologists (with 4 and 7 years of experience in interpreting central nervous system MR images, respectively) independently carried out these segmentations through the image segmentation software ITK-SNAP (version 3.8.0, http://itksnap.org). After manual delineation was accomplished, volumetric calculations of the segmented VOIs were performed accurately via the integrated statistical analysis features within ITK-SNAP (Fig. 2 (**B**)).

#### Histopathological analysis

Tumor tissues resected during surgery were fixed in 4% buffered formalin and subsequently embedded in paraffin. The paraffin-embedded tissues were sectioned for histopathological and immunohistochemical analyses. Specifically, 4 μm thick sections were cut from the main tumor mass and stained with hematoxylin and eosin (H&E). The glioma type and grade were determined according to the 2016 WHO classification for cases prior to August 2021 and according to the 2021 WHO classification for cases thereafter [21, 22]. The expression of proteins such as glial fibrillary acidic protein (GFAP), alpha-thalassemia/mental retardation syndrome x-linked (ATRX), and oligodendrocyte transcription factor 2 (Olig-2) in tumors was detected via immunohistochemistry. Sanger second-generation sequencing was used to detect mutations in isocitrate dehydrogenase (IDH)1/2 and telomerase reverse transcriptase (TERT), O6-methylguanine-DNA methyltransferase (MGMT) methylation, epidermal growth factor receptor (EGFR) amplification, and chromosome 1p/19q codeletion [23]. To evaluate tumor cell proliferation, Ki-67 immunohistochemical staining was conducted, with the Ki-67 labeling index defined as the percentage of Ki-67-positive nuclei relative to the total number of malignant cells [24, 25].

### Statistical analysis

Statistical analyses were conducted using SPSS statistical software (Version 28, IBM, Armonk, New York, United States, https://www.ibm.com/analytics/spssstatistics-software) and R software (Version 4.2.3, https://www.R-project.org/). Descriptive statistics were used to summarize the demographic and clinical characteristics of all the subjects. The normality of continuous variables was assessed via the Shapiro‒Wilk test. For normally distributed data, an independent samples t test or one-way ANOVA followed by the least significant difference (LSD) post hoc test was performed to compare imaging and clinical characteristics between groups. For nonnormally distributed data, the Wilcoxon rank-sum test or the Kruskal‒Wallis test combined with Dunn’s post hoc test was applied. Differences in the ALPS indices of the bilateral cerebral hemispheres of all the subjects were tested via paired t tests. Categorical variables were analyzed via Pearson’s chi-square test. Univariate linear regression was used to assess the correlation between the ALPS index and both demographic and glioma characteristics, and significant variables were included in multivariate regression to detect independent factors. The relationships between quantitative parameters, such as the ALPS index and CSF volume, were evaluated via Spearman’s rank correlation coefficient. A *p* value of less than 0.05 was considered statistically significant. All the statistical tests were two-tailed.

## Results

### Study population characteristics

**Fig. 3:**
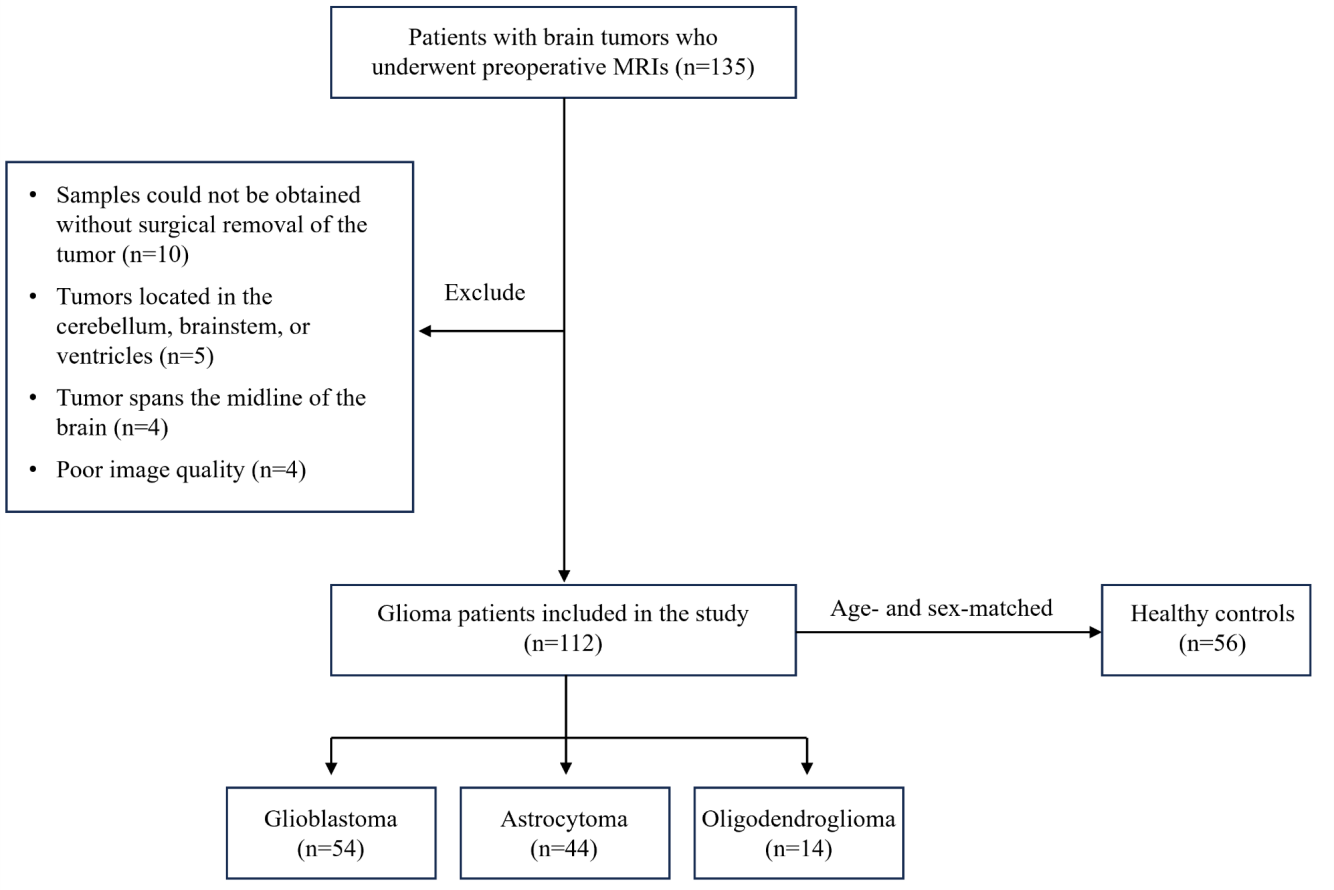
Flow chart of the study sample. In this investigation, 135 patients with brain tumors underwent MRI scanning. After screening by the exclusion criteria, 112 patients with gliomas were included in this study. In addition, 56 age- and sex-matched healthy controls were recruited

Our study included a total of 168 individuals, with 112 individuals diagnosed with glioma (mean age 52.8 ± 13.1 years, including 49 females) and a control group of 56 healthy subjects (mean age 53.4 ± 10.7 years, with 27 females). The demographic and clinical profiles of the study participants are summarized in Table 1, and the process of patient recruitment and classification is depicted in Fig. 3. Preoperative MRI scans were conducted on all glioma patients, followed by tumor resection within three days of imaging.

**Table 1:** Clinical characteristics of glioma patients and healthy controls

Characteristic	Patients (*n* = 112)	Health Control (*n* = 56)	*P*
Age, years	52.8 ± 13.1	53.4 ± 10.7	0.789
Gender			0.501
Male	63 (56.2)	29 (51.8)	
Female	49 (43.8)	27 (48.2)	
Intracranial tumor location			
Left hemisphere	60	N/A	
Right hemisphere	42	N/A	
Both hemispheres	10	N/A	
Grade		N/A	
LGGs	35		
HGGs	77		
IDH status		N/A	
Mutant	36		
Wildtype	76		
Ki-67 (%)	20.0 (25.0)	N/A	
Classification		N/A	
astrocytoma	44		
oligodendroglioma	14		
glioblastoma	54		

Age is presented as mean ± standard deviation; Ki-67 is shown as median (interquartile range). All other values represent the number of individuals, with percentages in parentheses

### Comparative analysis of neuroimaging biomarkers

**Fig. 4:**
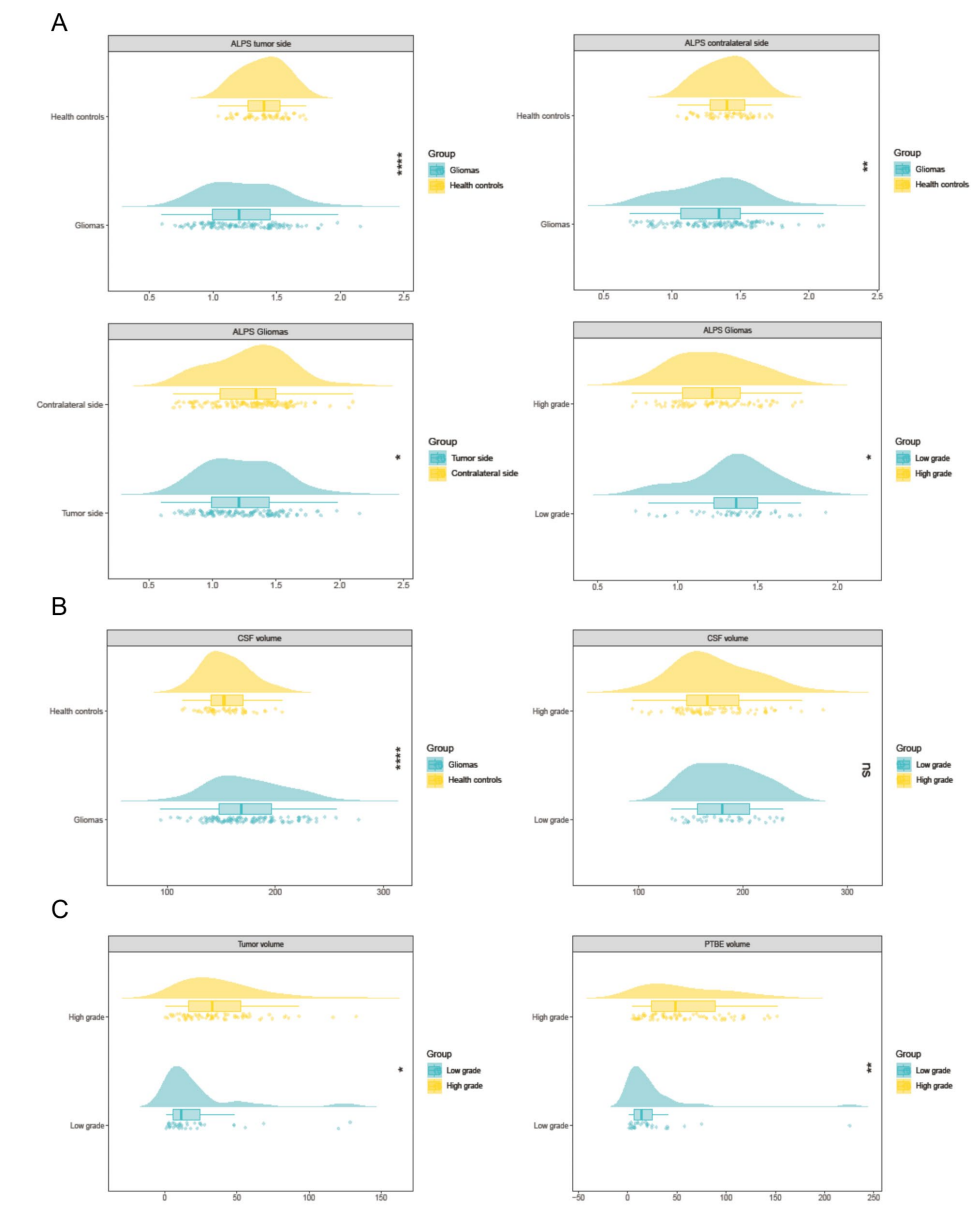
Intergroup Comparative Raincloud Plots of Quantitative MRI Data. (**A**) Intergroup comparative analysis of the ALPS index. (**B**) Comparative analysis of cerebrospinal fluid volume between groups. (**C**) Comparative analysis of tumor and PTBE volumes between high- and low-grade gliomas. * *p* < 0.05, ** *p* < 0.01, *** *p* < 0.001, ns = not statistically significant

The comparative analysis of neuroimaging biomarkers yielded significant findings. Compared with healthy controls, glioma patients demonstrated a notably lower mean ALPS index, calculated as the average of both hemispheres (1.266 ± 0.258 vs. 1.395 ± 0.174, *p* < 0.001). Additionally, an increase in CSF volume was detected in the glioma group relative to the control group (174.53 ± 34.89 cm³ vs. 154.25 ± 20.89 cm³, *p* < 0.001). The DTI-ALPS index in the hemisphere affected by the tumor was significantly lower than that in the contralateral hemisphere (1.233 ± 0.297 vs. 1.299 ± 0.296, *p* = 0.016), with both sides showing lower values than healthy controls did (*p* < 0.001, *p* = 0.009) (Supplementary Table 2). Compared with low-grade gliomas, high-grade gliomas had a lower DTI-ALPS index (*P* = 0.013) and greater tumor volume and PTBE volume (all *p* < 0.001), and no statistically significant difference in CSF volume was observed between the two groups (*p* = 0.394). There were no statistically significant differences in neuroimaging biomarkers between IDH wild-type and IDH-mutant tumors (all *p* > 0.05). A comparison of neuroimaging markers across glioma types revealed that glioblastomas had larger tumor volumes and lower ALPS indices than astrocytomas and oligodendrogliomas did (*p* = 0.017 and *p* = 0.004, respectively) (Table 2; Fig. 4). Intergroup comparisons for different tumor subgroups are shown in Supplementary Fig. 1.

**Table 2:** Between-group comparisons of CSF, tumor, and PTBE volumes; DTI-ALPS index

Groups	DTI-ALPS	*P*	CSF volume (cm^3^)	*P*	Tumor volume (cm^3^)	*P*	PTBE volume (cm^3^)	*P*
		**< 0.001**		**< 0.001**		/		/
Glioma	1.266 ± 0.258		174.53 ± 34.89		29.77(36.79)		37.48(65.11)	
Health control	1.395 ± 0.174		154.25 ± 20.89		/		/	
		**0.013**		0.394		**< 0.001**		**< 0.001**
LGG	1.345 ± 0.261		178.28 ± 32.25		11.76(33.03)		15.00(27.39)	
HGG	1.222 ± 0.246		172.48 ± 36.30		32.89(37.94)		49.98(63.75)	
		0.594		0.658		0.800		0.411
IDH-WT	1.261 ± 0.252		176.66 ± 37.75		27.59(32.42)		30.62(51.38)	
IDH-MUT	1.290 ± 0.289		173.23 ± 31.72		30.62(39.42)		24.86(86.97)	
		**0.017**		0.597		**0.004**		0.054
astrocytoma	1.333 ± 0.286	^**a**^ **0.024**	172.44 ± 33.55		22.24(41.65)	^**a**^ **0.043**	19.74(50.28)	
oligodendroglioma	1.378 ± 0.242	^**b**^ **0.017**	168.30 ± 34.17		17.76(22.85)	^**b**^ **0.002**	31.73(91.43)	
glioblastoma	1.204 ± 0.220	^c^0.563	178.15 ± 37.77		35.14(33.98)	^c^0.873	43.79(53.01)	

Bold text indicates statistical significance. ^a^ glioblastoma vs. oligodendroglioma, ^b^ glioblastoma vs. astrocytoma, ^c^ oligodendroglioma vs. astrocytoma; LGG, low-grade glioma; HGG, high-grade glioma; DTI-ALPS, the Diffusion Tensor Imaging analysis along the perivascular space; PTBE, peritumoral brain edema; IDH, isocitrate dehydrogenase; WT, wild type; MUT, mutation

### Correlation analysis of glioma characteristics with the ALPS index

**Fig. 5:**
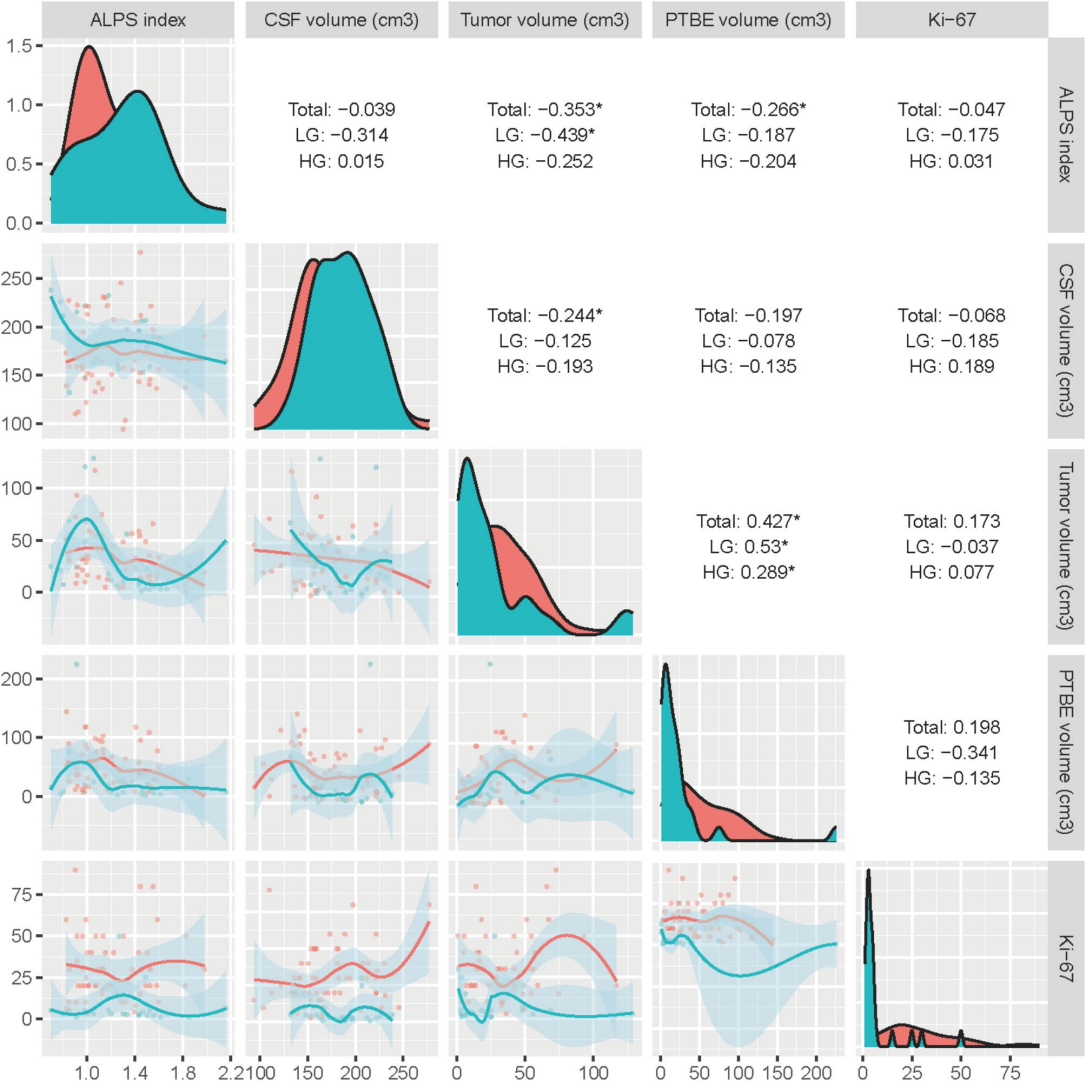
Correlation plots of the ALPS index, brain tumor volume, PTBE volume, and cerebrospinal fluid volume. The upper triangles display Spearman correlation coefficients, indicating the strength and direction of the relationships. The lower triangles feature scatter plots with fitted lines and confidence intervals, illustrating the trends. The diagonal shows the probability density distributions of the variables. Total, overall correlation; LGG, low-grade glioma; HGG, high-grade glioma. Significance levels: *, *p* < 0.05; **, *p* < 0.01; ***, *p* < 0.001

Univariate linear regression analysis revealed significant correlations between the ALPS index and certain demographic and tumor characteristics, including age (β = -0.228, *p* = 0.011), tumor grade (β = 0.222, *p* = 0.015) and Olig-2 expression (β = -0.284, *p* = 0.003). However, in the multivariate regression analysis, tumor grade was the only significant independent factor for the glioma ALPS index (β = -0.244, *p* = 0.011) (Supplementary Table 3). Furthermore, correlation analysis further illuminated the relationships among the ALPS index, tumor volume, and PTBE volume. A significant negative correlation existed between the ALPS index and tumor volume (*r* = 0.353, *p* < 0.001), with this association being particularly pronounced in low-grade gliomas (*r* = 0.439, *p* = 0.026). Additionally, the ALPS index was negatively correlated with PTBE volume (*r* = -0.266, *p* = 0.015). The positive correlation between tumor volume and PTBE volume (*r* = 0.427, *p* < 0.001) was consistent across both high-grade and low-grade gliomas (Fig. 5, Supplementary Table 4).

## Discussion

This prospective study provides novel insights into the complex relationship between gliomas and CSF dynamics by evaluating the ALPS index and volumetric parameters in glioma patients. Significant correlations between the ALPS index, tumor volume, and PTBE volume highlight the intricate interplay between tumor burden, glymphatic dysfunction, and cerebral edema.

Our findings revealed a significantly lower ALPS index in glioma patients than in healthy controls, suggesting impaired glymphatic function [26]. This finding is consistent with the findings of previous animal studies [27]. The glymphatic system plays a crucial role in clearing metabolic waste from the brain via perivascular pathways, facilitated by the movement of CSF and interstitial fluid [28]. Tumor-induced alterations in the brain microenvironment, including the disruption of perivascular spaces and compromised BBB integrity, likely contribute to this dysfunction [29]. Previous studies have shown that brain tumors can disrupt normal fluid dynamics through mechanical compression and vascular dysregulation [30, 31]. This impairment of the glymphatic system may exacerbate the accumulation of toxic metabolites, promoting tumor progression and contributing to the cognitive decline often observed in glioma patients [32, 33]. Furthermore, the ALPS index was lower in the tumor-affected hemisphere than in the contralateral hemisphere and on both sides than in healthy controls, indicating that tumors can directly affect glymphatic function in the affected hemisphere. This localized impairment may cause metabolic disturbances that extend beyond the immediate tumor region, potentially affecting neurological function in the contralateral hemisphere as well.

The inverse correlation between the ALPS index and both tumor volume and PTBE volume highlights the significant impact of tumor burden on glymphatic function and cerebral fluid balance. Larger tumors are typically associated with more extensive PTBE due to increased BBB permeability and subsequent extravasation of plasma components into the brain parenchyma [34]. This edema increases intracranial pressure, disrupts normal fluid clearance pathways, and further impairs glymphatic function.

The association of high-grade gliomas with a significantly lower ALPS index than low-grade gliomas suggests that tumor aggressiveness exacerbates these disturbances. Interestingly, while CSF volume was greater in glioma patients than in healthy controls, no significant difference was observed between high-grade and low-grade gliomas. This finding suggests that the increase in CSF volume may reflect a generalized disruption in intracranial fluid dynamics due to the presence of a tumor rather than being directly related to tumor aggressiveness.

The absence of significant differences in neuroimaging biomarkers between IDH wild-type and IDH mutant tumors is notable. These findings suggest that the IDH mutation status may not significantly influence glymphatic function or cerebral fluid dynamics in this context, which contrasts with the findings of several previous reports [26, 35]. IDH mutations are well-established markers of prognosis in gliomas, with IDH-mutant tumors generally associated with a better overall prognosis than IDH-wild-type tumors. This mutation often indicates favorable biological behavior and a different tumor microenvironment [36]. However, our results highlight the complexity of this relationship, suggesting that the IDH mutation status may not be as directly linked to glymphatic function as previously thought. Further research is warranted to explore the underlying mechanisms and validate these findings.

Additionally, our study revealed that glioblastomas presented significantly larger tumor volumes and lower ALPS indices than astrocytomas and oligodendrogliomas did, reflecting their aggressive characteristics and consequently severe glymphatic dysfunction. Moreover, glioblastomas mainly originate from astrocytes, which are important components of the PVS and express AQP4 at their endfeet, which play a key role in facilitating the exchange of CSF and ISF. The aggressive nature of glioblastomas may lead to more extensive destruction of the perivascular space and a reduction in AQP4-mediated fluid exchange, resulting in an observed reduction in the ALPS index.

Multivariate analysis identified tumor grade as an independent factor associated with the ALPS index, which is consistent with previous findings [35], underscoring the pivotal role of tumor aggressiveness in glymphatic dysfunction. Owing to their increased invasiveness and larger tumor burden, high-grade gliomas may further disrupt perivascular spaces and fluid clearance pathways. Additionally, the reduced expression of vascular endothelial growth factor-C (VEGF-C) observed in high-grade glioblastomas may hinder the formation and expansion of meningeal lymphatic vessels, further impairing glymphatic drainage [37, 38]. Although age and Olig-2 were not identified as independent predictors of the ALPS index in multivariate analysis, their correlation with the ALPS index remains clinically relevant. Numerous studies have demonstrated a strong negative correlation between age and glymphatic function [39, 40]. Olig-2 expression is similar in adult low-grade astrocytomas and oligodendrogliomas but is lower in IDH-wildtype glioblastomas [41, 42]. Therefore, higher Olig-2 expression may indicate lower tumor aggressiveness, resulting in less disruption to the glymphatic system and a higher ALPS index. The significant correlations between the DTI-ALPS index, tumor volume, and PTBE volume offer valuable insights into the underlying pathophysiological mechanisms. These findings suggest that as tumors grow and exert more pressure on surrounding tissues, they not only contribute to edema but also interfere with the ability of the brain to clear waste products effectively. This creates a vicious cycle in which impaired waste clearance further promotes tumor-associated pathology, potentially accelerating disease progression.

The results of this study offer promising clinical insights for glioma treatment. The observed link between tumor burden, PTBE, and glymphatic dysfunction suggests that targeting glymphatic impairment could be beneficial. Therapeutic strategies such as drugs that enhance the function of AQP4 and VEGF-C or cervical deep lymphovenous anastomosis (LVA) could promote the clearance of toxic substances, as well as the migration and activation of lymphocytes, such as CD8 T cells, thus contributing to the reduction in cerebral edema as well as an enhanced antitumor response [37, 43, 44]. Therefore, early detection of reduced glymphatic function through a lower ALPS index may guide more aggressive interventions for high-grade gliomas, potentially improving patient outcomes by slowing tumor progression and mitigating cognitive decline.

Nonetheless, several limitations should be considered in this study. First, the relatively small sample size, particularly when different glioma grades were compared, may limit the statistical power of our findings. Future studies should aim to include larger cohorts to validate these results. Second, glioma classification followed the 2016 WHO criteria before August 2021 and the 2021 criteria thereafter, introducing potential heterogeneity in tumor grading that may impact the interpretation of glioma subtypes’ effects on glymphatic function. Third, our study relies primarily on imaging data to infer glymphatic dysfunction, and the absence of molecular or histological validation leaves room for further investigation. The incorporation of advanced molecular imaging techniques and the exploration of markers such as AQP4 could provide deeper insights into glymphatic alterations in glioma patients. Finally, the cross-sectional design of this study does not allow for the assessment of dynamic changes in the glymphatic system over time. Longitudinal studies would be invaluable in understanding whether surgical or medical interventions can restore glymphatic function and improve clinical outcomes.

## Conclusion

In conclusion, this study highlights the complex interactions among glioma growth, cerebral edema, and glymphatic dysfunction, with significant implications for understanding glioma pathophysiology. The DTI-ALPS index could serve as a potential biomarker for glymphatic function impairment, guiding future therapeutic strategies aimed at alleviating tumor-related cerebral edema and enhancing waste clearance. Further research is warranted to explore these mechanisms and their clinical applications.

## Electronic supplementary material

Below is the link to the electronic supplementary material.

**Supplementary Table 1**: Summary of MRI Scanning Parameters. **Supplementary Table 2**: Intergroup comparison of DTI-ALPS indices in both hemispheres. **Supplementary Table 3**: Univariate and multivariate linear regression analysis of ALPS index with demographic and tumor characteristics. **Supplementary Table 4**: Correlation analysis of CSF, tumor, and PTBE volumes, DTI-ALPS index and Ki-67 expression levels. **Supplementary Figure 1**: Raincloud plots for intergroup comparison of MRI parameters for different subtypes of gliomas. (**A**) Comparative intergroup analysis of IDH-WT and IDH-MUT. (**B**) Intergroup comparative analysis of glioblastoma, astrocytoma, and oligodendroglioma. *P < 0.05, **P < 0.01, ***P < 0.001, ns = not statistically significant

## Data Availability

No datasets were generated or analysed during the current study.

## Abbreviations

AQP4: Aquaporin-4
BBB: Blood–brain barrier
CSF: Cerebrospinal fluid
DTI-ALPS: Diffusion tensor imaging analysis along the perivascular space
ICP: Intracranial pressure
IDH: Isocitrate dehydrogenase
MRI: Magnetic resonance imaging
PTBE: Peritumoral brain edema
PVS: Perivascular spaces
VEGF-C: Vascular endothelial growth factor-C
VOIs: Volumes of interest
