# Supplementary material for: Glymphatic system dysfunction and cerebrospinal fluid retention in gliomas: evidence from perivascular space diffusion and volumetric analysis
Source: Cancer Imaging. 2025 Apr 7;25:51. doi: 10.1186/s40644-025-00868-y (PMC11974089; doi:10.1186/s40644-025-00868-y)
Supplement: Supplementary file 1 — Supplementary Table 1: Summary of MRI Scanning Parameters. Supplementary Table 2: Intergroup comparison of DTI-ALPS indices in both hemispheres. Supplementary Table 3: Univariate and multivariate linear regression analysis of ALPS index with demographic and tumor characteristics. Supplementary Table 4: Correlation analysis of CSF, tumor, and PTBE volumes, DTI-ALPS index and Ki-67 expression levels. Supplementary Figure 1: Raincloud plots for intergroup comparison of MRI parameters for different subtypes of gliomas. (A) Comparative intergroup analysis of IDH-WT and IDH-MUT. (B) Intergroup comparative analysis of glioblastoma, astrocytoma, and oligodendroglioma. *P < 0.05, **P < 0.01, ***P < 0.001, ns = not statistically significant [file 40644_2025_868_MOESM1_ESM.docx]

**Supplementary materials**

**Supplementary Table 1.** Summary of MRI Scanning Parameters.

| Parameter/Sequence | 3D TIW GRE | 3D T2W FLAIR | T2W FSE | DTI |
| --- | --- | --- | --- | --- |
| TR (ms) | 7.2 | 6000 | 5385 | 5116 |
| TE (ms) | 3.1 | 430 | 95 | 74.2 |
| Flip angle | 10° | 180° | 180° | 90° |
| Slice thickness (mm) | 1.0 | 1.0 | 5 | 4 |
| Slice number | 256 | 256 | 23 | 40 |
| FOV (mm2) | 240×256 | 240×256 | 200×230 | 224×224 |

TR, repetition time; TE, echo time; FOV, field of view.

**Supplementary Table 2.** Intergroup comparison of DTI-ALPS indices in both hemispheres.

| Parameter | Glioma | |  |  | Health control | | | *^c^P* | *^d^P* |
| --- | --- | --- | --- | --- | --- | --- | --- | --- | --- |
|  | Tumor side | Contralateral side | *^a^P* |  | Left | Right | *^b^P* |  |  |
| ALPS index | 1.233±0.297 | 1.299±0.296 | 0.016 |  | 1.408±0.206 | 1.382±0.175 | 0.233 | <0.001 | 0.009 |

DTI-ALPS, the Diffusion Tensor Imaging analysis along the perivascular space

**Supplementary Table 3.** Univariate and multivariate linear regression analysis of ALPS index with demographic and tumor characteristics.

| Parameter | Univariable Linear Regression | |  | Multivariable Linear Regression | |
| --- | --- | --- | --- | --- | --- |
|  | β | *P* |  | β | *P* |
| Age | -0.228 | **0.011** |  | -0.131 | 0.202 |
| Sex | -0.170 | 0.059 |  |  |  |
| Tumor grade | 0.222 | **0.015** |  | -0.244 | **0.011** |
| Tumor type | 0.230 | **0.015** |  | 0.060 | 0.637 |
| IDH | 0.099 | 0.283 |  |  |  |
| Ki-67 | -0.140 | 0.144 |  |  |  |
| GFAP | 0.096 | 0.316 |  |  |  |
| Olig-2 | 0.284 | **0.003** |  | 0.149 | 0.968 |
| P53 | -0.180 | 0.059 |  |  |  |
| ATRX | 0.062 | 0.519 |  |  |  |
| TERT | -0.215 | 0.036 |  |  |  |
| MGMT | -0.049 | 0.634 |  |  |  |
| EGFR | -0.186 | 0.145 |  |  |  |
| 1p/19q | 0.032 | 0.760 |  |  |  |

IDH, Isocitrate Dehydrogenase; GFAP, Glial Fibrillary Acidic Protein; Olig-2, Oligodendrocyte Transcription Factor 2; ATRX, Alpha Thalassemia/Mental Retardation Syndrome X-Linked; TERT, Telomerase Reverse Transcriptase; MGMT, O6-Methylguanine-DNA Methyltransferase; EGFR, Epidermal Growth Factor Receptor

**Supplementary Table 4.** Correlation analysis of CSF, tumor, and PTBE volumes, DTI-ALPS index and Ki-67 expression levels

| Variable_X | Variable_Y | Group | r | P |
| --- | --- | --- | --- | --- |
| CSF volume | ALPS index | Total | -0.039 | 0.729 |
|  |  | Low grade | -0.314 | 0.118 |
|  |  | High grade | 0.015 | 0.910 |
| Tumor volume | ALPS index | Total | **-0.353** | **0.001** |
|  |  | Low grade | **-0.439** | **0.026** |
|  |  | High grade | -0.252 | 0.059 |
| PTBE volume | ALPS index | Total | **-0.266** | **0.015** |
|  |  | Low grade | -0.187 | 0.359 |
|  |  | High grade | -0.204 | 0.128 |
| Ki-67 | ALPS index | Total | -0.047 | 0.675 |
|  |  | Low grade | -0.175 | 0.394 |
|  |  | High grade | 0.031 | 0.821 |
| Tumor volume | CSF volume | Total | -0.244 | 0.027 |
|  |  | Low grade | -0.125 | 0.540 |
|  |  | High grade | -0.193 | 0.151 |
| PTBE volume | CSF volume | Total | -0.197 | 0.075 |
|  |  | Low grade | -0.078 | 0.706 |
|  |  | High grade | -0.135 | 0.315 |
| Ki-67 | CSF volume | Total | -0.068 | 0.542 |
|  |  | Low grade | -0.185 | 0.365 |
|  |  | High grade | 0.189 | 0.160 |
| PTBE volume | Tumor volume | Total | **0.427** | **0.000** |
|  |  | Low grade | **0.530** | **0.006** |
|  |  | High grade | **0.289** | **0.030** |
| Ki-67 | Tumor volume | Total | 0.173 | 0.118 |
|  |  | Low grade | -0.037 | 0.856 |
|  |  | High grade | 0.077 | 0.569 |
| Ki-67 | PTBE volume | Total | 0.198 | 0.072 |
|  |  | Low grade | -0.341 | 0.088 |
|  |  | High grade | -0.135 | 0.318 |

Bold text indicates statistical significance. CSF, cerebrospinal fluid; PTBE, peritumoral brain edema; ALPS, analysis along the perivascular space.


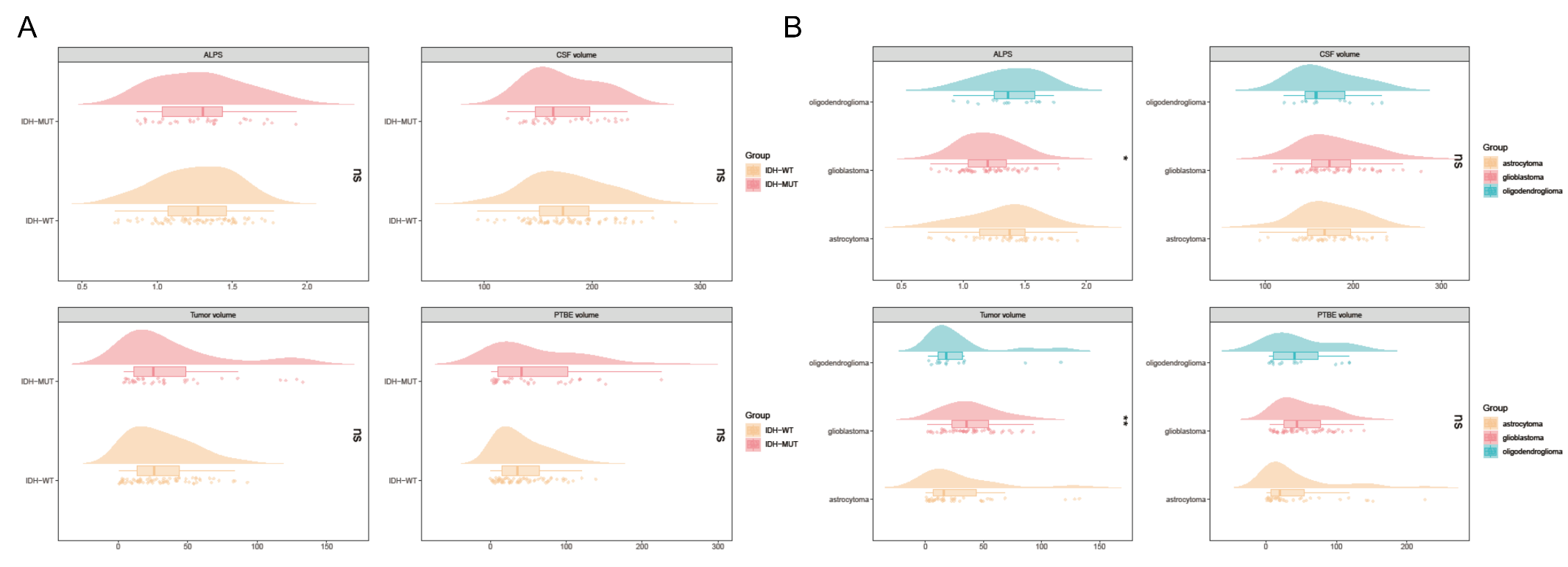


**Supplementary Figure 1.** Raincloud plots for intergroup comparison of MRI parameters for different subtypes of gliomas. (A) Comparative intergroup analysis of IDH-WT and IDH-MUT. (B) Intergroup comparative analysis of glioblastoma, astrocytoma, and oligodendroglioma. *P < 0.05, **P < 0.01, ***P < 0.001, ns = not statistically significant.
